# Lung function changes from childhood to adolescence: a seven-year follow-up study

**DOI:** 10.1186/s12890-015-0028-9

**Published:** 2015-04-03

**Authors:** Pavilio Piccioni, Roberta Tassinari, Aurelia Carosso, Carlo Carena, Massimiliano Bugiani, Roberto Bono

**Affiliations:** Unit of Respiratory Medicine, National Health Service, ASL TO2, Torino, Italy; Department of Public Health and Pediatrics, University of Torino, Via Santena 5 bis, 10126 Torino, Italy; Ospedale Maria Vittoria - ASL TO2, Torino, Italy

**Keywords:** Longitudinal study, Lung function, Adolescents, Air pollution, Environmental tobacco smoke

## Abstract

**Background:**

As part of an investigation into the respiratory health in children conducted in Torino, northwestern Italy, our aim was to assess development in lung function from childhood to adolescence, and to assess changes or persistence of asthma symptoms on the change of lung function parameters. Furthermore, the observed lung function data were compared with the Global Lung Function Initiative (GLI) reference values.

**Methods:**

We conducted a longitudinal study, which lasted 7 years, composed by first survey of 4–5 year-old children in 2003 and a follow-up in 2010. Both surveys consisted in collecting information on health by standardized SIDRIA questionnaire and spirometry testing with FVC, FEV_1_, FEV_1_/FVC% and FEF_25–75_ measurements.

**Results:**

242 subjects successfully completed both surveys. In terms of asthma symptoms (AS = asthma attacks or wheezing in the previous 12 months), 191/242 were asymptomatic, 13 reported AS only in the first survey (early transient), 23 had AS only in the second survey (late onset), and 15 had AS in both surveys (persistent). Comparing the lung function parameters observed with the predicted by GLI only small differences were detected, except for FVC and FEF_25–75_, for which more than 5% of subjects had Z-score values beyond the Z-score normal limits. Furthermore, as well as did not significantly affect developmental changes in FVC and FEV_1,_ the decrease in FEV_1_/FVC ratio was significantly higher in subjects with AS at the time of follow-up (late onset and persistent phenotypes) while the increase in FEF_25–75_ was significantly smaller in subjects with persistent AS (p < 0.05).

**Conclusions:**

The GLI equations are valid in evaluating lung function during development, at least in terms of lung volume measurements. Findings also suggest that the FEF_25–75_ may be a useful tool for clinical and epidemiological studies of childhood asthma.

## Background

The prevalence of childhood pulmonary diseases, especially bronchial asthma, is increasing worldwide [Global Initiative for Asthma (GINA) 2012, http://www.ginasthma.org/]: lung disorders in children [1,2] are most frequently of obstructive type and usually limited to the intrathoracic-intrapulmonary airways [3].

Reliable information of lung function would greatly benefit clinical assessment and patient follow-up. Studies on respiratory function tests in children and adolescents have been published [4], and specific criteria have been proposed for acceptable maximal expiratory flow volume (MEFV) and other reference values [5,6]. Findings arising from many of these studies have been assembled to generate the reference lung function equations proposed by the Global Lung Function Initiative (GLI) [7-9] of the European Respiratory Society. The Global Lung Project in 2012 (http://www.lungfunction.org/tools/90-equations-and-toolsml. The) published multi-ethnic reference equations for spirometry that span all-ages. However, these equations are until today not enough tested on the field, especially in children.

While lung development is a continuous process during childhood, lung function is dependent on age, gender, height, and ethnicity [10]. Both lung volume and forced expiratory volumes increase during this period, but not at the same rate [11]. Forced vital capacity (FVC) is affected by changes in muscular strength, by the shape and stiffness of the thorax, and by the number and size of alveoli in the lungs. Airflow, as measured for example by the FEV_1_, is also affected by the caliber of the airways, and by lung and airways elasticity. In childhood, the FVC increases more rapidly than the FEV_1_, leading to falls in the FEV_1_/FVC ratio, but these trends are temporarily reversed in adolescence [11] before continuing to decrease during adult life. Measurement of lung function is important for the evaluation of physical development and the presence of disease but few studies evaluated the effects of respiratory symptoms and their possible changes over time on transformation of lung function parameters.

In 2003, we studied a group of children, age 3–6 years, attending kindergarten in Torino, Italy [5]. We collected data on respiratory function (by spirometry) and respiratory health (by standardized questionnaire) [12]. In 2010, we carried out a follow-up study of the same group of children.

The aims of the present study were: 1) to establish if our data, drawn from a Northern Italian population, conforms with the GLI reference values; 2) to assess changes in lung function and to evaluate the effects of asthma symptoms (AS) on changes of lung function parameters in children during the transition to adolescence.

## Methods

### Study population

As part of a research project funded by the Piedmont Regional Council (northwest Italy) focusing on the effects of environmental pollution in preschoolers, in 2003 we studied 960 children, aged 3–6 years, drawn from 18 kindergarten schools located in Torino (6.700 inhabitants/km^2^, 240 m a.s.l.), an urbanized Italian city with almost 900.000 inhabitants. This initial cohort was whittled down to 766 children as some declined to participate or spirometry testing was deemed invalid. To update the database in 2010, we again contacted the individuals studied in 2003. The demographic information provided by the parents, together with the information within the 2003 consent forms, allowed us to reconstruct a database of 573 children, now drawn from 20 secondary schools located in Torino. Each child was given the same the identification code in the 2003 and 2010 surveys to facilitate data comparison between surveys. Since the subjects were underage, during a public meeting in both the two occasions, parents and teachers were informed on the objective of this study. A written informed consent was signed and delivered by each the participants’ parents. Thus, the participation of all the subjects did not occur until after informed consent was obtained. However, the local Ethics Committee “San Luigi Gonzaga Hospital” (previously named “ASL TO2”) has expressed a favorable opinion with practice number 826/13/08.

### Questionnaire

Child health information was collected in the 2003 and 2010 surveys using the same standardized SIDRIA (Italian Studies on Respiratory Disorders in Children and the Environment) questionnaire [12,13] compiled by the parents. The questionnaire was administered aiming to check the presence of respiratory symptoms and related risk factors.

### Spirometry

Written informed consent was obtained from the parents prior to both 2003 and 2010 measurements. Spirometry was carried out in the morning during school activities. Height (measured with a stadiometer), weight and body mass index (BMI) (computed as weight/height^2^) of each child were also recorded. Pulmonary function was measured using a turbine-based Masterscope Rotary Jaeger spirometer with subjects standing and wearing a nose-clip. In 2003, we organized the children into small groups and, with playful communication, we explained how to carry out the test. All the tests were performed using special incentive spirometry software (“blowing out candles” software). Spirometry testing was performed in a similar manner in 2010, without the use of “blowing out candles” software. In both surveys, each child recorded 3–6 MEFV curves within a 10–15 min interval. Subjects with only one acceptable measurement were excluded from the analysis. The exclusion criteria adopted in 2003 are specified elsewhere. In particular we have considered not acceptable the manoeuvres with: a) a sub-maximal expiratory effort in which a peak expiratory flow (PEF) was not clearly determined (i.e. in presence of flat or rounded curves), or with slow rise of PEF (top of the curve to the right) [14,15]; b) evidence of cough or glottis closure [6]; c) an expiration time lesser than 0.5 seconds [15]; d) an abrupt end of expiration effort (presence of a sharp drop or cessation in flow from a point in which the flows where >25% of PEF) [14,15]. Furthermore, children with reported skeletal anomalies or lung diseases, other than asthma, were excluded [5]. In 2010, the exclusion criteria adopted were in accordance with the current guidelines [10]. For subjects who reported actual acute symptoms or taking drugs for respiratory disorders, the examination was rescheduled after adequate washout period (at least 24 hours) or symptom remission.

### Statistical methods

Lung function test results were used only if valid in both surveys. Descriptive statistics on both occasions were performed and reported for all subjects. All analysis were performed by means of STATA® 12 statistical package (StataCorp College Station, Texas 77845 USA). In the analyses, asthma symptoms (AS) where defined as referred presence of asthma attacks or episodes of wheezing in the previous 12 months.

The subjects were categorized as following: *asymptomatic* if AS was absent in both surveys, *early transient* with AS in the first but not in the second survey, *late onset* with AS in the second survey only and *persistent* if AS was presents in both surveys. Following the results of Box-Cox regression, Linear transformations were applied when indicated to correct for heteroscedasticity and deviation from normal distribution.

To assess how our data for FEV_1_, FVC, FEV_1_/FVC% and FEF_25–75_ conformed with the GLI reference values, the GLI reference values were computed for each subject by means of the GLI-2012 Desktop Software for Data Sets vs 1.3.4, available on the web site http://www.lungfunction.org/tools/90-equations-and-tools/196-obtain-software.html.

The means of observed values in asymptomatic as measured in each survey and values predicted by GLI were compared by means of *t*-test for paired data. The Z-score were also computed and the 5^th^ and 50^th^ percentiles were reported.

To measure the effects of time, anthropometric variables and symptoms on changes in lung function parameters over the time, annual changes were computed as the difference between the parameters from the second and the first survey, divided by years of follow-up. Annual changes were used in a set of multiple regressions as dependent variable, and gender and time varying covariates (anthropometric values: height, weight, BMI) and AS in both occasions as predictors, using asymptomatic subjects group as reference. Marginal means with confidence intervals (C.I.) at 95% were calculated from predictions at mean values of covariates and averaging over symptoms at the follow-up.

## Results

In 2010, we traced and contacted 573 of the 766 children studied in 2003; of these, 174 declined to participate in the second survey. Consequently, the follow-up survey had 399 participants, mean age 11.8 years, drawn from 20 primary and secondary schools in Torino. Of these 399 subjects, 242 (60.3% males) adequately performed lung function tests in both surveys, and only these subjects were included in this analysis. The decision to participate in the surveys was not influenced by anthropometric variables or symptoms. Table 1 reports the main anthropometric characteristics and results of lung function tests in the two surveys.

**Table 1:** **Characteristics and findings of lung function test of the 242 subjects at the first (2003) and second (2010) survey**

	**FEMALE**			**MALE**			**TOTAL**		
	**2003**	**2010**	**Δ/year**	**2003**	**2010**	**Δ/year**	**2003**	**2010**	**Δ/year**
	**Mean**	**Mean**	**Mean**	**Mean**	**Mean**	**Mean**	**Mean**	**Mean**	**Mean**
	**(sd)**	**(sd)**	**(sd)**	**(sd)**	**(sd)**	**(sd)**	**(sd)**	**(sd)**	**(sd)**
**AGE**	4.5	11.7	7.34	4.5	11.8	7.23	4.5	11.8	7.30
	(0.7)	(0.6)	(0.48)	(0.6)	(0.7)	(0.43)	(0.7)	(0.7)	(0.46)
**HEIGHT (cm)**	110.3	150.1	5.3	111.7	150.7	5.5	111.1	150.4	5.5
	(6.2)	(8.5)	(0.7)	(6.3)	(8.8)	(0.7)	(6.3)	(8.7)	(0.7)
**WEIGHT (Kg)**	18.8	42.6	3.4	20.0	44.8	3.3	19.5	43.9	3.3
	(3.2)	(9.3)	(1.2)	(3.8)	(11.3)	(1.0)	(3.6)	(10.5)	(1.0)
**BMI%**	15.4	18.6	0.41	15.9	19.0	0.44	15.7	18.8	0.43
	(1.8)	(3.7)	(0.59)	(2.0)	(4.9)	(0.46)	(1.9)	(4.4)	(0.54)
**FVC(ml)**	1054	2647	221	1144	2842	231	1105	2756	227
	(239)	(508)	(55)	(226)	(533)	(55)	(236)	(530)	(55)
**FEV**_**1**_ **(ml)**	1048	2293	176	1127	2426	177	1093	2367	177
	(224)	(420)	(44)	(211)	(460)	(52)	(220)	(447)	(48)
**FEV1%/FVC**	96.6	85.3	−1.7	96.3	83.7	−1.8	96.4	84.3	−1.8
	(4.4)	(7.4)	(1.1)	(4.1)	(6.0)	(0.9)	(4.3)	(6.5)	(1.0)
**FEF**_**25–75**_ **(ml/s)**	1561	2718	159	1735	2706	131	1660	2711	143
	(385)	(728)	(88)	(949)	(731)	(162)	(761)	(729)	(136)

Among the 242 subjects, 28 (11.6%) had AS in the first survey, 38 (17%) had AS in the second survey whereas 191 subjects did not have AS in either survey. Otherwise, from a longitudinal point of view, 13 children reported AS in the first but not in the second survey (early transient), 23 reported AS only in the second survey (late onset), and 15 had AS at the time of both surveys (persistent) (Table 2).

**Table 2:** **Distribution of asthma symptoms (AS = Asthma or wheezing in the previous 12 months) on two observations**

	**No symptoms**	**Early transient**	**Late onset**	**Persistent**
N	191	13	23	15
%	78.93	5.37	9.5	6.2

The Table 3 shows the results of comparison of observed with the GLI predicted values for FEV_1_, FVC, FEV_1_/FVC % and FEF_25–75_ in asymptomatic subjects. The FEV_1_, FVC observed values were lower than predicted in both surveys while FEV_1_/FVC% and FEF_25–75_ were lower than predicted in the second occasion only; the differences were significant at 5% level. However, the differences, as shown by the 95^th^ percentile of Z-score, were within the range of normal variation of the reference values except for of the values of FVC in both occasions and FEF_25–75_ in the second one, where more than five percent (but less than 10%) of the subjects fell below the lower predicted Z-score.

**Table 3:** **Means and changes by year of lung function parameter observed and predicted from GLI and their differences for asymptomatic subjects, compared with** ***t*** **-test for paired data**

		**2003**		**2010**		**Δ/year**	
		**Mean**	**p**	**Mean**	**p**		**p**
**FEV**_**1**_ **(ml)**	**Observed**	1107.4		2435.9		180.4	
	**Predicted**	1123.2		2497.5		*188.3*	
	**δ**	−15.8	**NS**	−61.6	*	−7.9	*
	**95% CI of δ**	(−32.8 - +5.6)		(−103.4 --19.8)			
	**Z-score 50**_**tile**_	−0.138		−0.203			
	**Z-score 5**_**tile**_	−1.583		−1.548			
	***lln***	*879.5*		*2017.8*			
**FVC (ml)**	**Observed**	1120.7		2824.1		233.4	
	**Predicted**	1216.0		2876.4		227.8	
	**δ**	−95.2	**	−52.3	**	*5.6*	**
	**95% CI of δ**	(−116.2 - -74.3)		(−91.9 - -12.7)			
	**Z-score 50**_**tile**_	−0.645		−0.113			
	**Z-score 5**_**tile**_	**−1.954**		−1.468			
	***lln***	*943.9*		*2329.9*			
**FEV**_**1**_ **/FVC (%)**	**Observed**	96.6		85.5		−1.57	
	**Predicted**	92.9		87.1		−0.8	
	**δ**	3.7	**	−2.4	**	−0.77	**
	**95% CI of δ**	(+3.0 - +4.3)		(−2.4 - -0.8)			
	**Z-score 50**_**tile**_	0.961		−0.207			
	**Z-score 5**_**tile**_	−0.905		−1.664			
	***lln***	*81.6*		*76.3*			
**FEF**_**25–75**_ **(ml/s)**	**Observed**	1621.3		2766.9		*152.8*	
	**Predicted**	1602.6		2955.8		185.2	
	**δ**	18.7	**NS**	−269.6	**	−32.3	**
	**95% CI of δ**	(−25.0 - +62.3)		(−269.6 - -108.2)			
	**Z-score 50**_**tile**_	0.045		−0.306			
	**Z-score 5**_**tile**_	−1.357		−1.912			
	***lln***	*967.0*		*1954.3*			

Legend:

δ: Observed. Predicted by GLI.

Δ: (Differences between the 2 occasions/Years of follow-up).

* p < 0.05 ** p < 0.01.

CI = Confidence interval.

Z-Score = (standard deviation scores from regression Equation).

50_tile_ = Median 5_tile_ = fifth percentile.

lln = lower limit of normality of predicted values.

**Figure 1:**
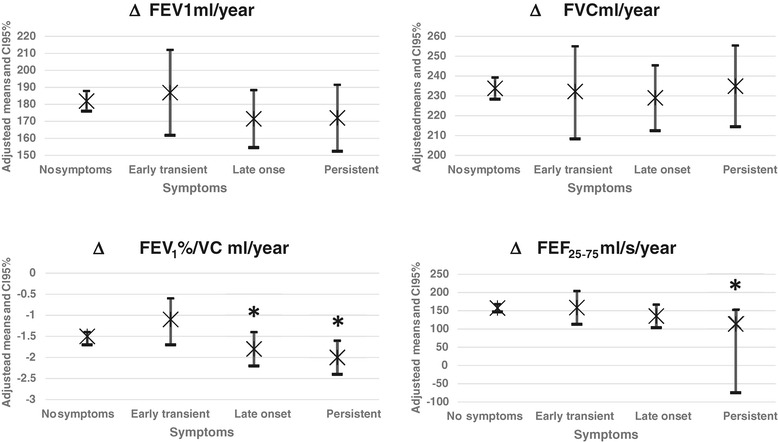
**Annual mean changes of lung function parameters (adjusted for sex and age at the first observation) with CI 95%, by symptom groups.** Note: The * indicates the groups with significant difference at 5% level (p < 0.05) versus asymptomatic group

The marginal means (and C.I. 95%) of the lung function parameters (adjusted for height and gender) measured in the first survey and their annual changes are shown (Table 4), stratified according to AS status. The Figure 1 shows the adjusted means of annual changes by symptom group: the lung function levels measured during the first survey (adjusted for sex and height) were not significantly different among symptoms status groups. However, annual changes seemed to be affected by AS. When volumes were taken into account, AS of symptomatic subjects did not induced significant changes in FVC, FEV_1_ if compared to asymptomatic subjects. Comparing the symptomatic subjects to asymptomatic ones, the decrease in the FEV_1_/FVC% was significantly higher in subjects with AS in the second survey (late onset and persistent), but not in transient. Finally, FEF_25–75_ showed a significantly higher decrease in subjects with persistent AS (*p* < 0.05).

**Table 4:** **Lung Function parameters in the first survey and annual changes (mean and C.I. 95%) adjusted for height and gender with multiple regression analysis**

		**2003**	**Δ/year**	**P<**
		**Mean**	**Mean**	**model**
		**(C.I.)**	**(C.I.)**	
**FEV**_**1**_ **(ml)**	**No symptoms**	1108.8	181.9	**NS**
		(1087.6-1129.9)	(176.0 -187.9)	
	**Early transient**	1122.3	186.9	
		(1032.2-1212.4)	(161.8 - 212.1)	
	**Late onset**	1105.7	171.5	
		(1045.31 -166.0)	(154.6 - 188.4)	
	**Persistent**	1100.2	172.0	
		(1030.5 - 1169.9)	(152.4 - 191.5)	
**FVC (ml)**	**No symptoms**	1121.4	233.8 (228.4 - 239.3)	**NS**
		(1100.6 - 1142.1)		
	**Early transient**	1159.3	232.2	
		(1072.7-1245.8)	(208.4 - 255.9)	
	**Late onset**	1134.6	229.0	
		(1072.3 - 1197.0)	(212.5 - 245.4)	
	**Persistent**	1151.5	234.9	
		(1074.3 - 1228.7)	(214.5 - 255.4)	
**FEV**_**1**_ **/FVC (%)**	**No symptoms**	96.5	−1.5	*
		(95.9 - 97.2)	(−1.7 - -1.4)	
	**Early transient**	94.2	−1.1	
		(91.5 - 97.0)	(−1.7 - -0.6)	
	**Late onset**	96.7	−1.8	
		(94.9 - 98.5)	(−2.2 - -1.4)	
	**Persistent**	95.9	−2.0 *	
		(93.8 - 98.0)	(−2.4 - -1.6)	
**FEF**_**25–75**_ **(ml/s)**	**No symptoms**	1621.9	157.4	***
		(1580.6 - 1663.1)	(146.8 - 168.0)	
	**Early transient**	1531.5	158.6	
		(1353.4 - 1709.6)	(113.0 - 204.1)	
	**Late onset**	1570.3	135.5 *	
		(1447.4 - 1693.1)	(104.0 - 166.9)	
	**Persistent**	1506.4	113.7 **	
		(1354.2 - 1658.6)	(74.7 - 152.7)	

- * P < 0.05.

- ** P < 0.01.

Δ: (Differences between the 2 occasions/Years of follow-up)

Note: the statistical significativity in the last column is for the model; the * in the cell reports the significativity of the difference of the mean compared with the asymptomatic subjects mean.

## Discussion

The interpretation of functional parameters in the transition from childhood to adolescence was highlighted by the multiplicity of reference values and the discontinuity between age groups. The Global Lung Initiative (GLI) equations are the first global multiethnic reference equations for spirometry that span all ages and which seem to have solved this problem. A major limitation of any reference equation is that it is based on a cross-sectional snapshot of a population composed of individuals at different ages, but the age differences do not necessarily reflect the individual changes over time [16-18]. However, our results suggest that, at least for the age and time range examined in this study, the predictions of the GLI reference equations are a good approximation of the changes in lung function observed over time and adequately describe pulmonary function in growing subjects.

When we compared the values observed in asymptomatic subjects with the predicted by the GLI, the means were consistently different only for FVC and FEF_25–75_. Although, for the most part of subjects these differences were within the range of normal variation of the GLI references values, they may indicate that some regional differences are possible, even among the same ethnic group. Thus, at least for FEF_25–75_, we recommend the use of regional data; at least until a proper longitudinal equation including individuals who have completed their lung growth.

Factors having a negative impact on the age-related growth of pulmonary function in children and adolescents may result in a lower maximally attained level of pulmonary function and perhaps in an earlier onset in decline of pulmonary function. Therefore, these factors potentially increase the risk of subsequently developing both reversible and irreversible obstructive pulmonary diseases [17-19].

Numerous follow-up studies in children with asthma have consistently shown that more severe respiratory symptoms in childhood predict reduced lung function in early adulthood [20,21]. In the mean time, epidemiological studies of childhood asthma are highlighting the causality between asthma and deficits in lung function, although the characteristics of growth in pulmonary function from childhood to adulthood are not completely clarified yet [22-26]. Determining whether the loss of lung function and asthma are, respectively, the cause or the effect of one another is crucial for the prevention of asthma and for our understanding of the origins of this important disease. The contradictory results can be due to differences in the frequency of assessment, cohort retention rates, and the use of quantitative measurements [27,28].

In the present study, the longitudinal analysis of a cohort of children examined in 2003 and 2010 showed that the presence of wheezing disorders (wheezing or asthma attack) at the time of both measurements (persistent phenotype) was associated with a smaller increase in forced flows (particularly FEF_25–75_). In contrast, changes in the FEV_1_/FVC% (and FEV_1_ and FEV_0.5_, although not statistically significant at 5% level) was affected by the presence of symptoms in the second measurement.

The FEV_1_ (and the FEV_1_/FVC%), which is considered to be reproducible and to represent an appropriate measure of airway obstruction, often shows normal values even in children with symptoms of uncontrolled asthma. In this regard, asthmatic patients may have ventilatory defects in the presence of normal FEV_1_ [29-32].

The FEF_25–75_ is a more sensitive marker of symptomatic asthma than the FEV_1_ in children [31] and in adults [32]. The middle volume flow rates, measured by the FEF_25–75_, is theoretically less effort-dependent than the FEV_1_, also because FEF_25–75_ is a measurement of the “small airways” patency [32,33] and does not include high flows in the lung volume. On the contrary, the guidelines of the American Thoracic Society and the GLI do not suggest that the assessment of FEF_25–75_ could play a significant role in the measurement of airflow obstruction [10,34]. The coefficient of variation of instantaneous flow is quite large, which partly explains their unsatisfactory performance in clinical decision-making. Moreover, the bronchodilators can affect the natural transformations of flows and, consequently, the values of FVC, making them no longer comparable with one another.

On the other hand, the FEF_25–75_ measured in children provides, compared to the FEV_1_, additional information about clinical status and airway inflammation. Furthermore, the FEF_25–75_ is well correlated with bronchodilator responsiveness in asthmatic children with normal FEV_1_, and the FEF_25–75_ is associated with increased childhood asthma severity and morbidity [35].

Middle volumes flows rates, particularly the FEF_25–75_, seem to be a more sensitive indicator of small airway disease. Despite concerns of test-to-test variability and the lack of a clearly defined normal range, our findings suggest that the FEF_25–75_ is clinically relevant in children with asthma and could distinguish between subjects with symptoms that persist over time from subjects with transient or late-onset symptoms. However, in large and statistically powerful studies, that can compensate for the variability, the FEF_25–75_ can be a useful tool to detect mean differences between groups with different clinical characteristics. In these circumstances, the benefits of the increased physiological sensitivity of the FEF_25–75_ remain.

### Study potential limitations

A possible limitation of this study could either be the relevant loss of subjects to follow-up because we were not able to trace them or because the subjects declined to repeat lung function testing or were not able to perform it. A further limitation can be due to impossibility to measure FEV1 in some children 3–4 years [5] aged because their expiratory time was less than 1 second: this produced a loss of subjects for longitudinal comparison.

Due to the initial estimate, the sample size in the symptomatic strata could be too small to show as statistically significant effects. However, although still inaccurate the estimation and the relative confidence intervals are quite informative to assess the direction of the effects.

## Conclusions

Our results confirm the validity of the GLI reference equations in evaluating lung function during growth, at least as regards the dynamic volumes. Furthermore, our results highlight the usefulness of FEF_25–75_ measurement as a tool for clinical and epidemiological studies of asthma in children.

## Abbreviations

GLI: Global Lung function Initiative
SIDRIA: Italian Studies on Respiratory Disorders in Children and the Environment
ALS: Asthma Like Symptoms
FVC: Forced Vital Capacity
FEV_1_: Forced Expiratory Volume in the 1st second
FEV_1_/FVC: Forced Expiratory Volume related to some portion of the FVC
FEF_25–75_: Forced Espiratory Flow 25–75% of FVC
